# Effect of the submandibular push exercise using visual feedback from pressure sensor: an electromyography study

**DOI:** 10.1038/s41598-020-68738-0

**Published:** 2020-07-16

**Authors:** Sungwon Park, Joo Young Cho, Byung Joo Lee, Jong-Moon Hwang, Myunghwan Lee, Soo Yeon Hwang, KwanMyung Kim, Ki Hoon Lee, Donghwi Park

**Affiliations:** 1 Department of Rehabilitation Medicine, Daegu Fatima Hospital, Daegu, Republic of Korea; 2 Department of Rehabilitation Medicine, School of Medicine, Kyungpook National University, Kyungpook National University Hospital, Daegu, Republic of Korea; 3 Medical Device Development Center, Daegu-Gyeongbuk Medical Innovation Foundation, Daegu, Republic of Korea; 4 Graduate School of Creative Design Engineering, Ulsan National Institute of Science and Technology, Ulsan, Republic of Korea; 5 Mompyeonhan Rehabilitation Clinic, Daegu, Republic of Korea; 6 Department of Physical Medicine and Rehabilitation, Ulsan University Hospital, University of Ulsan College of Medicine, 877, Bangeojinsunhwando-ro, Dong-gu, Ulsan, 44033 Republic of Korea

**Keywords:** Medical research, Neurological disorders, Disability

## Abstract

We developed a new exercise method called the submandibular push exercise that can strengthen the suprahyoid muscle by inducing only the motion of the hyoid bone without neck flexion. In this study, we aimed to investigate and compare the muscle activity of the suprahyoid and infrahyoid muscles in the course of performing three different swallowing exercises. Twenty healthy participants and fifteen patients with dysphagia were recruited. Each participant consecutively performed three exercises: Shaker, CTAR, and submandibular push exercises. To investigate muscle activation, surface electromyography was performed on the suprahyoid, infrahyoid, and SCM muscles, during the exercises. Root mean square (RMS) was measured. In healthy participants, the submandibular push exercise showed a significantly higher RMS value in the suprahyoid and infrahyoid muscles than the Shaker and CTAR exercises using repeated ANOVA with Tukey's post hoc test (*p* < 0.05). In patients with dysphagia, the submandibular push and Shaker exercises showed significantly higher RMS value in the suprahyoid and infrahyoid muscles than the CTAR exercise. However, no significant difference was found between the submandibular push and Shaker exercises. In both healthy and patients with dysphagia, the mean RMS values of the SCM muscles during the submandibular push exercise were significantly lower than those during the Shaker exercise using repeated ANOVA with Tukey's post hoc test (*p* < 0.05). In conclusion, considering the relatively superior selectiveness in suprahyoid and infrahyoid muscle contraction, the submandibular push exercise using visual feedback from pressure sensor could be an efficient supplementary exercise to the conventional swallowing muscle exercises. However, further studies may be necessary to confirm the improvement in swallowing difficulty.

## Introduction

Swallowing is a complex sensorimotor process that involves the coordinated contraction and relaxation of the musculature located around the mouth, tongue, larynx, pharynx, and esophagus. Different levels of the central nervous system from the cerebral cortex to the medulla oblongata are also involved in normal swallowing process^1,2^. Among the numerous muscles that are involved, the importance of the suprahyoid and infrahyoid muscles has been investigated extensively in previous studies^2–4^. The suprahyoid muscle is known to move the hyoid bone in an anterosuperior direction, whereas the thyrohyoid muscle, one of the infrahyoid muscles, moves the larynx in an anterosuperior direction^2,5–8^. Other infrahyoid muscles, such as the sternohyoid, omohyoid, and sternothyroid muscles, act as a hyolaryngeal complex depressor^2,5–8^. In a previous study, the subsequent contractions of the suprahyoid and infrahyoid muscles are verified to accomplish the circular motion of the hyoid bone^5^. In addition, the infrahyoid muscles assist in the opening of the upper esophageal sphincter (UES) by the anterior movement of the hyoid bone^5^.

Various exercise maneuvers, such as the Shaker, tongue, and chin tuck against resistance (CTAR) exercises, have been used in clinical settings to strengthen the swallowing-related muscles^9–11^. The Shaker exercise consists of sustained and successive head lifts performed by the patient while in the supine position^11^. It was developed to strengthen the suprahyoid muscle, thereby helping in UES opening^11,12^. It is effective in restoring oral feeding in patients with pharyngeal dysphagia due to incomplete UES opening^10,11^. Moreover, the Shaker exercise significantly increases the anteroposterior diameter of the UES in elderly patients with and those without dysphagia; thus, patients with dysphagia exhibit a significant reduction in post-swallow aspiration^12,13^. Surface electromyography (sEMG) findings of the swallowing-related muscles taken while the patient performed the Shaker exercise provide evidence of fatigue in the suprahyoid muscle, indicating that it is physiologically affected by the Shaker exercise^13^. However, Yoshida et al.^14^ reported that the Shaker exercise may be too physically demanding for elderly patients with chronic disease. To overcome the limitation of the Shaker exercise, CTAR exercise, which can be performed with the patient seated in a chair, has been suggested in other studies^10,11,14^. In the CTAR exercise, the resistance is achieved by compressing an inflatable rubber ball or plastic bar between the chin and the manubrium sternum^10,11^. The CTAR exercise has been reported to significantly show greater maximum suprahyoid muscle sEMG values than the Shaker exercise^11^. On the other hand, Gao et al.^9^ reported that the CTAR and Shaker exercises showed similar effectiveness in improving swallowing function in patients with dysphagia.

The main kinetic motion of both the CTAR and Shaker exercises is head and neck flexion, which is not the main function of the suprahyoid muscle. Thus, considering the function of the suprahyoid and infrahyoid muscles (movement of the hyoid bone upward and downward), we thought of a new exercise maneuver, which does not include the motion of neck flexion, to possibly strengthen the suprahyoid muscle more selectively. We developed a new exercise method called the submandibular push exercise that may strengthen the suprahyoid muscle selectively by provoking only the motion of the hyoid bone and not neck flexion. In this study, we aimed to investigate and compare the muscle activity of the suprahyoid and infrahyoid muscles in the course of performing the Shaker, CTAR, and submandibular push exercises.

## Materials and methods

### Participants

This study was approved by the Institutional Review Board of Daegu Fatima Hospital (DFH19ORIO383) and informed consent has been obtained from the study participants. In this prospective case–control study conducted between August 2019 and February 2020, 35 adult participants (20 healthy participants and 15 patients with dysphagia) were initially recruited (Table 1). The healthy group consisted only of healthy subjects with no disease history that could cause dysphagia (stroke, spinal cord injury, or etc.) and no dysphagia. The patients with dysphagia had variable etiologies with at least one symptom of dysphagia, such as food sticking in throat, coughing when eating, globus sensation, drooling, having a weak or wet voice, and difficulty in chewing^15,16^. All patients had stable vital signs and were physically able to participate in the study. Patients with severe cognitive dysfunction (≤ 9 points of Mini-mental status examination (MMSE)) or serious psychiatric disorder, with upper extremity weakness that prevents from holding a device during exercise, and with other problems that limit the use of devices during exercise, as well as those aged less than 20 years were excluded^16^.

**Table 1:** Characteristics of participants.

	Healthy participants	Patients with dysphagia
Number	20	15
Sex ratio (M:F)	10:10	9:6
Age (years)	29.13 ± 5.694	70.20 ± 8.77
Duration (months)		4.57 ± 3.22
Cause of dysphagia		Hemispheric stroke (n = 10)
		(SICH = 1, infarction = 9)
		Brain stem stroke (n = 4)
		(Infarction = 4)
		TSAH (n = 1)
Symptoms of dysphagia		Protective cough with eating (n = 15)
		Food sticking in throat (n = 7)
		Drooling (n = 2)
		Having a wet or weak voice (n = 3)
		Globus sensation (n = 5)
		Difficulty chewing (n = 4)

Mean ± standard deviation, M:F, male:female; TSAH, traumatic subarachnoid hemorrhage; SICH, spontaneous intra-cerebral hemorrhage.

Food sticking, cough with eating, globus sensation, or diet change.

### Design

Three exercises (Shaker, CTAR, and submandibular push exercises) were consecutively performed by all participants. Patients were allowed to rest between each exercise to prevent muscle fatigue. Resting activation levels were recorded immediately prior to each exercise.

### Development of submandibular push exercise

**Figure 1:**
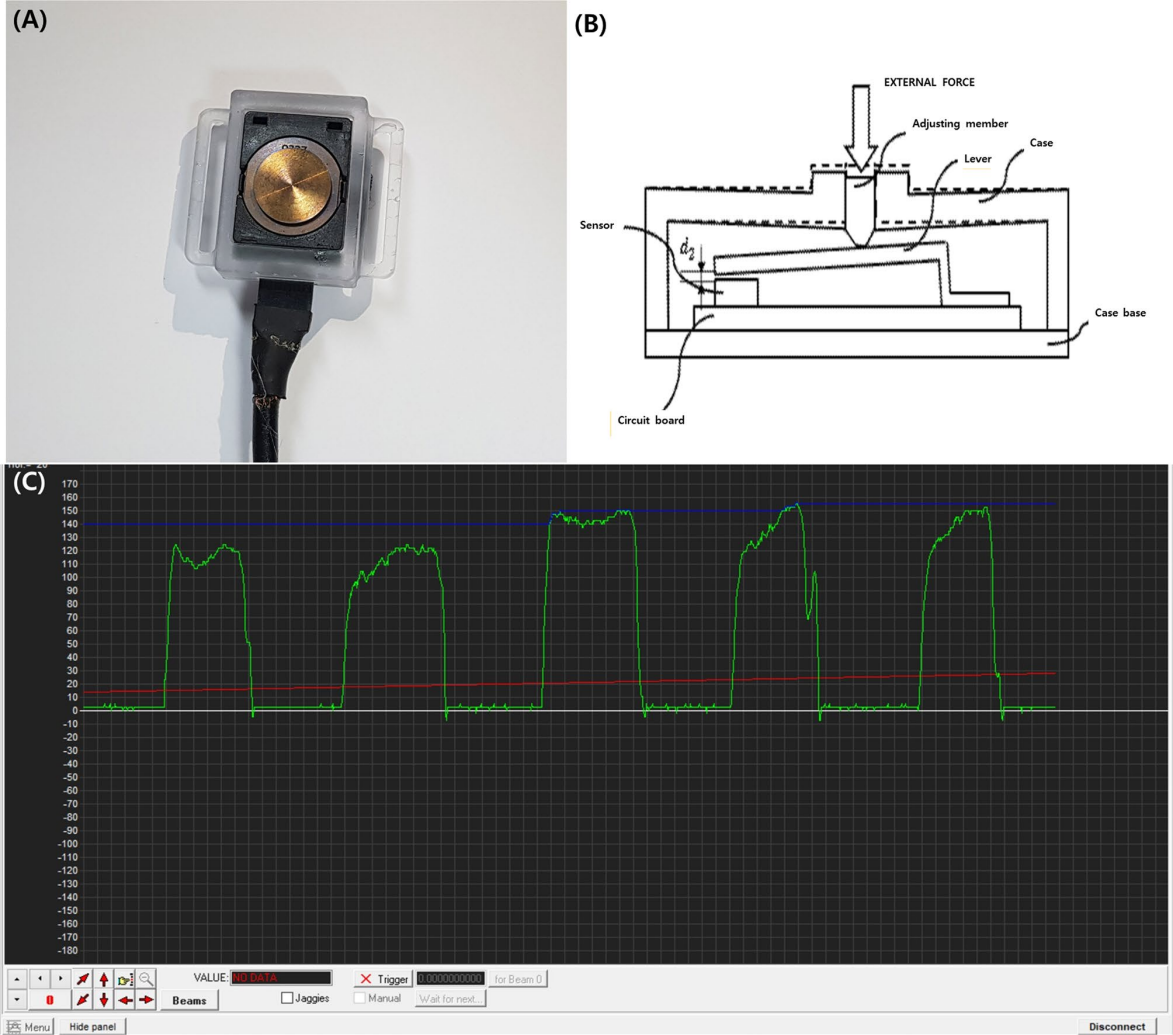
(**A**) Load cell pressure sensor. (**B**) View for explaining an operation mode of the force sensor using a displacement amplification mechanism according to the exemplary embodiment of the present invention. (**C**) Monitoring of pressure sensor during the submandibular push exercise, which used visual feedback.

Twelve healthy adult participants aged 21–39 years were recruited to evaluate the correlation between submandibular pressure and suprahyoid muscle activity. We investigated the correlation between submandibular pressure and suprahyoid muscle activity (Fig. 1) by using a pressure sensor (FS2050-0000-1500-G Load Cell; Te Connectivity, Switzerland) and sEMG under the submandibular muscles (suprahyoid muscle). Participants wore a headgear with pressure sensor under the suprahyoid muscles (Supplementary 1). They were instructed to increase the submandibular pressure without neck flexion.

To increase the submandibular pressure, participants were instructed to try to lower the hyoid bone by voluntary contraction of the infrahyoid muscle. When the hyoid bone was lowered, the suprahyoid muscle then started to contract eccentrically. In this process, the suprahyoid started to tighten by its eccentric contraction and place increased pressure on the pressure sensor under the suprahyoid muscles. Patients were asked to maintain an end-posture of the submandibular push exercise maneuvers to maintain the highest submandibular pressure using the visual feedback of the pressure sensor on the computer monitor. To reduce motion artifacts, sEMG data were recorded while maintaining this state (highest submandibular pressure). The root mean square (RMS) value was measured 10 times with 1 s as a section.

During this exercise, the maximal submandibular pressure (in the pressure sensor under the submandibular muscles) and maximal activity of the suprahyoid and infrahyoid muscles (in the sEMG) were recorded using an EMG device and software (Medelec Synergy; CareFusion Corporation, San Diego, CA).

Using the values of maximal submandibular pressure and maximal activity of the suprahyoid and infrahyoid muscles (RMS value), we investigated the activity of the swallowing muscles. We found that the values of the pressure sensor under the submandibular area had a significant correlation (Supplementary 2–4). Based on the significance of this result, the submandibular push exercise maneuver, which does not involve neck flexion motion, was developed.

### Procedure

Each participant was evaluated in a quiet room. All methods were carried out in accordance with relevant guidelines and regulations that comply with institutional, national, or international guidelines. The therapeutic benefits of the exercises were explained to the participants before they signed the informed consent form. The participants then completed a health questionnaire. The screening procedure ended with an oromotor examination. Finally, a brief explanation and demonstration of the three exercises were provided.

Each participant performed one trial of each of the three exercises in the following order (one trial consisted of 10 repetitions): (1) CTAR exercise, (2) submandibular push exercise, and (3) Shaker exercise. A 5-min rest period was provided between each exercise to prevent muscle fatigue. Patients were asked to maintain an end-posture of each exercise for 10 s. The 10-s duration was adapted from previous studies^11,14^. Participants were reminded to breathe and keep their mouths closed during all three exercises.

### Exercise maneuver

For the Shaker exercise, the participants were asked to lie in supine position and perform the following task: lift and hold the head up for 10 s. They were instructed to lift the head high enough to see their own feet without raising their shoulders^13^. For the CTAR exercise, the participants were asked to sit on a chair with their back upright and to press a plastic bar, a CTAR device (ISO-CTAR Device; Alternative Speech and Swallowing Solutions), which was placed under the chin by tucking the chin as hard as possible (Supplementary 1)^11^.

Before the start of the submandibular push exercise, participants underwent a 15-min trial session using visual feedback. The participants sat on a chair with their back upright and wore a headgear with pressure sensor under the submandibular area (Supplementary 1). They were then instructed to increase the submandibular pressure without neck flexion (Supplementary 1). To increase the submandibular pressure, participants were asked to try to lower the hyoid bone by voluntary contraction of the infrahyoid muscle. When the hyoid bone was lowered, the suprahyoid muscle then started to contract eccentrically^17^. In this process, the suprahyoid started to tighten by its eccentric contraction and place increased pressure on the pressure sensor under the suprahyoid muscles^17^. Using the visual feedback of the pressure sensor on the computer monitor, participants were taught to properly push the pressure sensor on the submandibular area (the pressure sensor that was placed under the suprahyoid muscles was pushed by bloating their submandibular area with their lips and teeth closed), and they were asked to press the pressure sensor as hard as possible without flexing their neck.(Fig. 1-C) (Supplementary 1). If the patients were well acquainted with the submandibular push exercise, they were asked to perform the submandibular push exercise in the same way without pressure sensor. Patients were instructed to maintain an end-posture of the three different exercise maneuvers, and the sEMG data were recorded. The sEMG signal was then measured 10 times with 1 s as a section.

### Electromyography

Multi-channel sEMG was performed during the three exercises using an EMG device and software (Medelec Synergy; CareFusion Corporation, San Diego, CA)^2,18^. sEMG was targeted for the suprahyoid muscles (mylohyoid and anterior belly of digastric muscles), infrahyoid muscles (thyrohyoid and sternothyroid muscles), and sternocleidomastoid (SCM) muscles^8^ (Supplementary 5). Ultrasound examination was performed to confirm the location of sEMG on targeted muscles. Using a 3–16- MHz linear ultrasound probe (Samsung Medison, Hongchun, Korea), the precise location of the muscle belly in each swallowing muscle was evaluated. Active surface electrodes were attached to the evaluated muscle belly, and reference surface electrodes were attached to the anterior surface of the mandible and clavicle^19^. Prior to the exercise, all participants rested for 5–10 min to adjust the sEMG.

Before the start of each exercise maneuver, participants underwent a 15-min trial session to obtain the RMS value at maximal voluntary contraction. Data were collected at a sampling rate of 50 kHz using Medelec Synergy with the following settings: low-frequency filter, 20 Hz; high-frequency filter, 1,000 Hz; sweep speed and gain, 1 s/div and 100 uV/div; and common mode rejection ratio, > 110 dB^2,18,20^. A custom-designed disposable pre-gelled 20-mm Ag/AgCl disc electrode (CareFusion, Höchberg, Germany) was used to measure EMG activity. Active electrodes were placed over the muscle belly of each swallowing muscle, and ground electrodes were positioned over an electrically silent area, generally the base of the mandible.

### Signal processing

As mentioned above, the RMS value was measured 10 times with 1 s as a section. We calculated the average RMS value of the swallowing muscles during 1 s by using EMG software (Medelec Synergy; CareFusion Corporation, San Diego, CA). A total of 10 RMS values were calculated in each exercise maneuver. Among the 10 RMS values (one second section) in each exercise maneuver, the highest RMS value was defined as the maximal RMS value. In addition, the average RMS value was calculated as the average of 10 RMS values (one-second section).

### Subjective feedback

After completing all three exercises, participants were given a 10-min rest. Subsequently, each participant answered the following questions: which of the three exercises was more strenuous, and which of the three exercises was more difficult to understand and properly perform.

### Sample size calculation

The maximal RMS value of the suprahyoid muscle (μV) was obtained from a pilot sample of 10 healthy participants. It was used to perform a two-tailed sample size calculation for the expected mean differences, with a standard deviation (SD) of 0.2, a significance level of 0.05, and a power of 80%. Accordingly, the required sample size for this study was at least 7 patients in each group.

### Statistical analysis

IBM SPSS version 21 (SPSS, Inc., Chicago, IL, USA) and PRISM software version 8.00 (GraphPad Software, Inc., San Diego, CA, USA) were used for the statistical analysis. To determine whether significant differences exist among the three different exercises, the maximum and mean RMS values for the 35 participants were compared using repeated ANOVA with Tukey's post hoc test. *p* < 0.05 was considered statistically significant.

## Results

### Participant characteristics

A total of 20 healthy subjects (10 males and 10 females) aged 21 to 39 years (mean = 29.13, SD = 5.69) and 20 patients with dysphagia (10 males and 10 females) aged 52 to 84 years (mean = 70.20, SD = 8.77) were recruited (Table 1). The causes of dysphagia were stroke (n = 14) and traumatic brain injury (n = 1). The duration of the diseases was 1 to 12 months (mean = 4.57, SD = 3.22) (Table 1).

### Comparison of muscle activation levels for the three exercises in healthy participants

**Figure 2:**
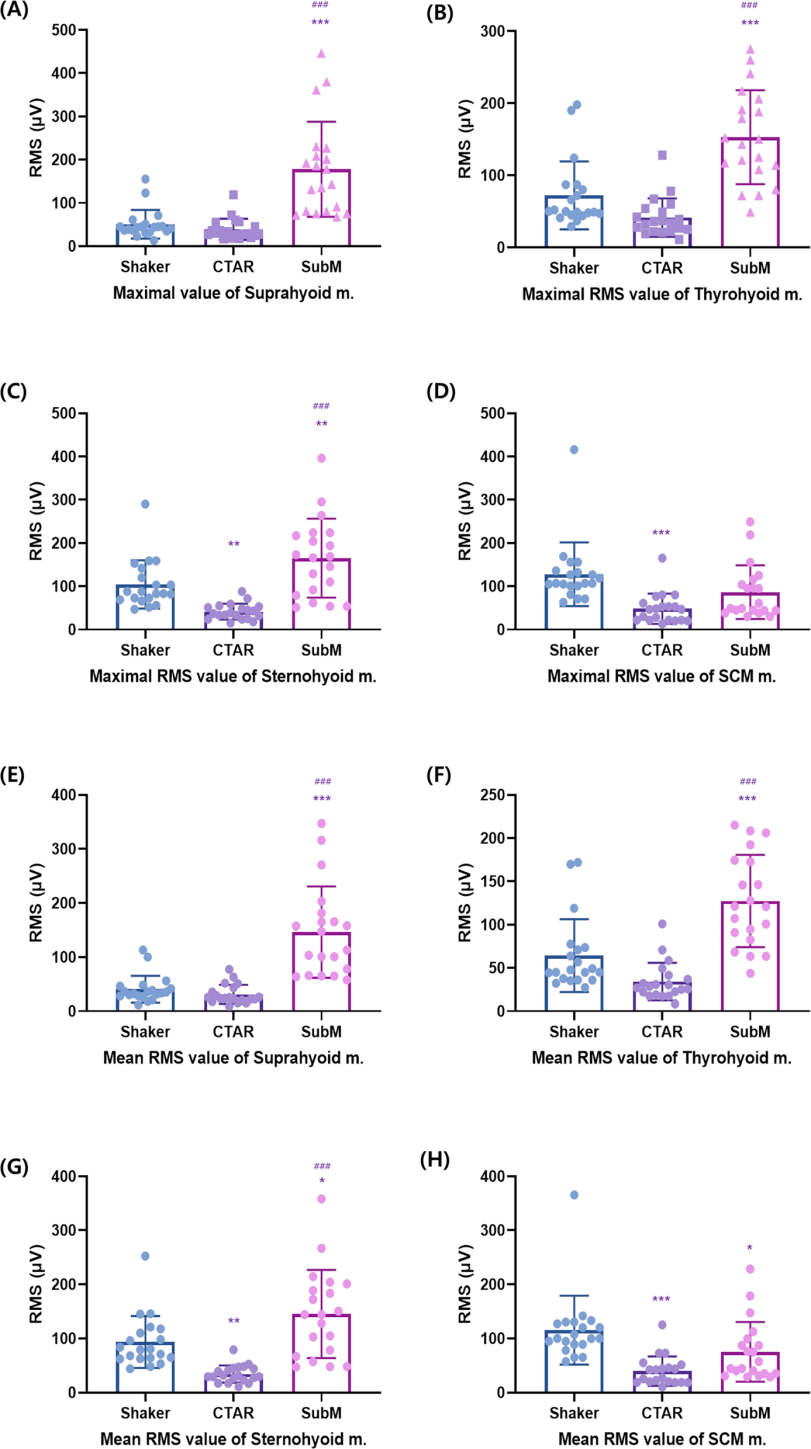
Maximal (**A**–**D**) and mean (**E**–**H**) RMS values of the suprahyoid, thyrohyoid, sternohyoid, and SCM muscles in healthy participants. Graphs were drawn using GraphPad Prism software 8.0. RMS, root mean square; SCM, sternocleidomastoid. vs. Shaker; *p < 0.05, **p < 0.01, ***p < 0.001, vs. CTAR; ^#^*p* < 0.05, ^##^*p* < 0.01, ^###^*p* < 0.001.

The submandibular push exercise showed a significantly greater increase in the maximal and mean RMS values of the suprahyoid muscles than the Shaker and CTAR exercises (*p* < 0.001) (Fig. 2 and Table 2). However, no significant difference was found between the Shaker and CTAR exercises (*p* ≥ 0.05). In the maximal and mean RMS values of the thyrohyoid muscles, the submandibular push exercise showed a significantly greater increase than the Shaker and CTAR exercises (*p* < 0.001), but no significant difference was found between the Shaker and CTAR exercises (*p* ≥ 0.05). The submandibular push exercise also showed a significantly larger increase in the mean RMS value of the sternothyroid muscle than the Shaker and CTAR exercises (*p* < 0.05). In addition, the maximal and mean RMS values of the thyrohyoid muscles during the Shaker exercise showed a larger increase than those during the CTAR exercise (*p* < 0.05). In the mean RMS value of the SCM muscle, the Shaker exercise had a significantly greater increase than the CTAR and submandibular push exercises (*p* < 0.05). Moreover, the mean RMS value of the SCM muscle during the submandibular push exercise had a higher increase than that during the CTAR exercise (*p* < 0.05) (Fig. 2 and Table 3).

**Table 2:** The maximal RMS value of the suprahyoid, thyrohyoid, sternohyoid, and SCM muscles in healthy participants and patients with dysphagia.

		Max suprahyoid RMS			Max thyrohyoid RMS			Max sternothyroid RMS			Max SCM RMS		
		Shaker (μV)	CTAR (μV)	SubM (μV)	Shaker (μV)	CTAR (μV)	SubM (μV)	Shaker (μV)	CTAR (μV)	SubM (μV)	Shaker (μV)	CTAR (μV)	SubM (μV)
HP	Mean	50.80	39.10	178.00*^#^	72.25	41.55	153.00*^#^	104.45	41.40*	165.15*^#^	135.00	51.36*	101.86
	SD	32.98	24.26	109.86	47.14	26.66	65.03	55.95	18.23	91.14	86.25	38.93	67.54
DP	Mean	81.73	37.48*	80.99^#^	92.60	46.19*	78.19	98.40	43.16*	80.29^#^	103.93	54.98*	59.05*
	SD	49.92	27.63	35.81	53.57	30.36	60.52	27.81	27.98	48.27	39.00	29.12	29.56

RMS, root mean square; SD, standard deviation; CTAR, chin tuck against resistance; SubM, submandibular push exercise; SCM, sternocleidomastoid; Max, maximum; HP, healthy participants; DP, dysphagic patients.

vs. Shaker, *p < 0.05; vs. CTAR, ^#^*p* < 0.05.

**Table 3:** The mean RMS value of the suprahyoid, thyrohyoid, sternohyoid, and SCM muscles in healthy participants and patients with dysphagia.

		Mean Suprahyoid RMS			Mean Thyrohyoid RMS			Mean Sternothyroid RMS			Mean SCM RMS		
		Shaker (μV)	CTAR (μV)	SubM (μV)	Shaker (μV)	CTAR (μV)	SubM (μV)	Shaker (μV)	CTAR (μV)	SubM (μV)	Shaker (μV)	CTAR (μV)	SubM (μV)
HP	Mean	40.79	31.15	146.29*^#^	64.25	34.21	127.36*^#^	93.42	34.32*	145.40*^#^	122.09	41.39*	88.77
	SD	24.84	17.56	84.60	42.07	21.70	53.28	47.94	15.76	81.48	74.53	29.85	60.30
DP	Mean	63.32	31.67*	70.85^#^	77.67	36.47^#^	67.75	88.45	38.41*	71.44^#^	93.23	43.42*	50.38*
	SD	32.00	20.32	36.67	29.25	20.19	51.40	24.82	24.96	51.75	33.15	19.91	23.30

RMS, root mean square; SD, standard deviation; CTAR, chin tuck against resistance; SubM, submandibular push exercise; SCM, sternocleidomastoid; Max, maximum; HP, healthy participants; DP, dysphagic patients.

vs. Shaker, *p < 0.05; vs. CTAR, ^#^*p* < 0.05.

### Comparison of muscle activation levels for the three exercises in patients with dysphagia

**Figure 3:**
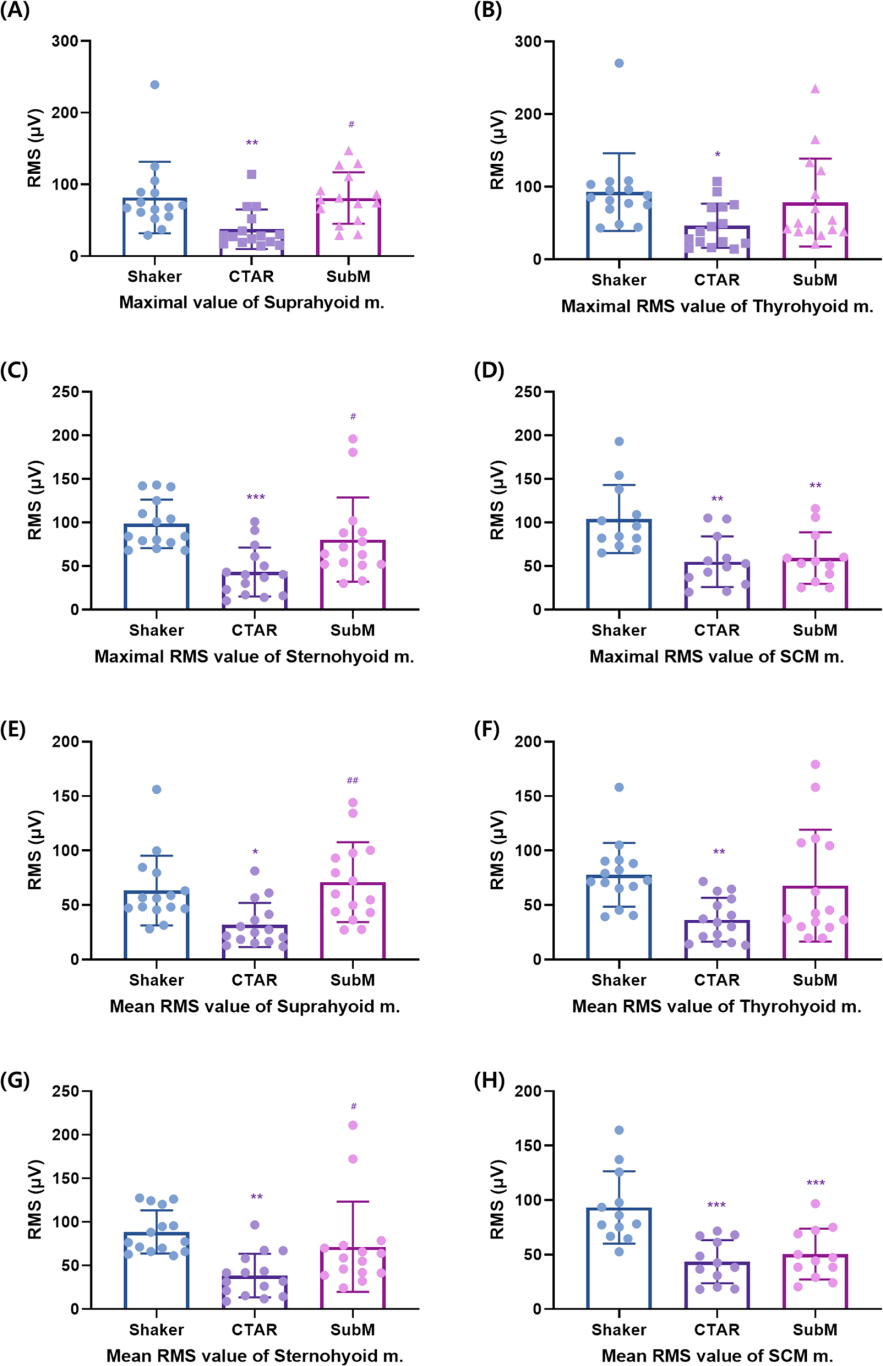
Maximal (**A**–**D**) and mean (**E**–**H**) RMS values of the suprahyoid, thyrohyoid, sternohyoid, and SCM muscles in patients with dysphagia. Graphs were drawn using GraphPad Prism software 8.0. RMS, root mean square; SCM, sternocleidomastoid. vs. Shaker; *p < 0.05, **p < 0.01, ***p < 0.001, vs. CTAR; ^#^*p* < 0.05, ^##^*p* < 0.01, ^###^*p* < 0.001.

The submandibular push exercise showed a significantly greater increase in the maximal and mean RMS values of the suprahyoid muscles than the CTAR exercise (*p* < 0.05) (Fig. 3 and Table 2). However, no significant difference was found between the Shaker and submandibular push exercises (*p* ≥ 0.05). In the maximal and mean RMS values of the thyrohyoid muscles, the submandibular push exercise showed a significantly greater increase than the CTAR exercise (*p* < 0.001). By contrast, significant difference was not observed between the Shaker and submandibular push exercises (*p* ≥ 0.05). The submandibular push and Shaker exercises showed a significantly greater increase in the maximal and mean RMS values of the sternothyroid muscle than the CTAR exercise (*p* < 0.05). However, no significant difference was found in the maximal and mean RMS values of the sternohyoid muscle between the Shaker and submandibular push exercises (*p* < 0.05). In the mean RMS value of the SCM muscle, the Shaker exercise showed a significantly greater increase than the CTAR and submandibular push exercises (*p* < 0.001). Significant difference was not found in the maximal and mean RMS values of the SCM muscle between the CTAR and submandibular push exercises (*p* < 0.05) (Fig. 3 and Table 3).

### Feedback from the participants

#### Feedback on the intensity of the exercise

From the subjective feedback based on the ternary decision, 85.7% (30/35) of the participants reported that Shaker exercise was most strenuous among the three exercises. 71.4% (25/35) of the participants reported that CTAR exercise was the least strenuous excise among the three exercises.

#### Feedback on the difficulty of how to perform the exercise

From the subjective feedback based on a ternary decision, 85.7% (30/35) of the participants reported that the submandibular push exercise was the most difficult to perform properly among the three exercises. About 88.5% (31/35) of the participants reported that the CTAR exercise was the easiest to perform among the exercises.

## Discussion

The aim of this study was to determine whether the submandibular push exercise is an effective swallowing exercise in terms of raising the sEMG activation level of the swallowing-related muscles, such as the suprahyoid and infrahyoid muscles. Two other well-known swallowing exercises were used for comparison^10,11,14^. The maximal and mean values of the sEMG measurements for each of the three exercises were recorded and analyzed. Participants were also asked to report the degree of intensity among the exercises and the difficulty of understanding how to perform the exercise.

Although a slight difference exists in muscle activation according to participant group, the sEMG results showed that the Shaker and submandibular push exercises were more powerful exercise methods for supra- and infrahyoid muscles compared with the CTAR exercise. The sEMG results for the muscle activation levels showed that the submandibular push exercise, using a visual feedback from the pressure sensor, resulted in a significantly greater activation of both supra- and infrahyoid muscles than the CTAR exercise. However, in the SCM muscle, the Shaker exercise showed a significantly higher RMS value than the CTAR and submandibular push exercises^14^. Our results were not consistent with those of previous studies, which reported the superiority of the CTAR exercise over the Shaker exercise in the muscle activation of the suprahyoid muscle. The inconsistent results might be attributed to the difference in tools used in the CTAR exercise. In previous studies^10,11^, a soft ball was used for the CTAR exercise, but the CTAR device (ISO-CTAR Device) was used in this study.

From the perspective of training specific swallowing muscles (supra- and infra-hyoid muscles), the Shaker exercise has a tendency to be inefficient. Although this exercise can induce the most powerful swallowing, it may be inefficient in training specific swallowing muscles. Moreover, in a subjective feedback, the Shaker exercise was reported to be the most strenuous compared with the other exercises. However, the submandibular push exercise using visual feedback had also been shown to provoke a powerful contraction of the swallowing muscles as much as the Shaker exercise. Moreover, considering the relatively lower contraction of the SCM muscle, the submandibular push exercise showed to be somewhat selective for contraction of the supra- and infrahyoid muscles. The CTAR exercise was the easiest exercise to perform in the subjective feedback, but was not very effective in contracting the swallowing muscle despite its relative selectivity for contracting the swallowing muscle. These findings demonstrate that the submandibular push exercise has an equivalent or greater impact than the CTAR or Shaker exercise on both the supra- and infrahyoid muscles without unnecessary activation of the SCM muscle.

One reason for the higher sEMG levels of the suprahyoid muscle during the submandibular push exercise might be that it induces the eccentric contraction of the suprahyoid muscle by depressing the hyoid bone in response to strenuous infrahyoid muscle contraction (Supplementary 1). When performing the submandibular push exercise, the hyoid bone was depressed due to strenuous infrahyoid muscle contraction (Supplementary 1). Therefore, the hyoid bone goes down by concentric contraction of the infrahyoid muscles; the suprahyoid is the eccentric contraction of the induced suprahyoid because the suprahyoid and infahyoid muscles are in balance state by pulling the hyoid bones up and down each other^2,5,6^. Moreover, since the submandibular push exercise does not involve neck flexion, unlike the Shaker and CTAR exercises, it might have prevented the inefficient co-contraction of the SCM.

Previous sEMG studies on the Shaker exercise showed that the SCM muscle fatigues earlier than the suprahyoid muscle^8,11^. This might limit the performance of the Shaker exercise and attainment of the exercise goal^11,13^. However, in our study, the RMS value of the SCM muscle was relatively lowered in the submandibular push exercise than that in the Shaker exercise. This indicates that the submandibular push exercise creates the least amount of unwanted SCM activation while properly strengthening the suprahyoid and infrahyoid muscles.

Despite the previous results, understanding how to perform the submandibular push exercise was the most difficult among three exercises. However, the exercise could be easily performed by all participants after providing proper exercise instruction using visual feedback from pressure sensor. Therefore, considering the pros and cons of the submandibular push exercise, supplementation of conventional exercises may be possible if sufficient and appropriate instruction is provided.

From an exercise-based therapeutic perspective, increased resistance during rehabilitation in abnormal muscle conditions promotes muscle adaptability, therefore inducing more effective functional recovery. Such may be a major goal in treating swallowing disorders. In addition, the high resistance load during exercise by using tools, such as elastic–plastic bar (Supplementary 6), that can provide resistance against muscle movement, can promote type I and type II muscle fiber remodeling in the skeletal muscle^21^. Therefore, the submandibular push exercise with a device that resists muscle movement may contribute more to the recovery of swallowing muscle function.

## Limitations

Our study has several limitations. First, we investigated the activation of the suprahyoid and infrahyoid muscles for one session of the three different exercises. Because at least 6–12 weeks of training is required to induce change in neuromuscular tissues, proving the actual change in contraction of the suprahyoid and infrahyoid muscles is difficult. However, muscle activation tends to be proportional to the amount of exercise, so the results of this study may reflect the possibility of actual swallowing muscle strengthening. Further study with more than 6 to 12 weeks of resistance training may be required to confirm the improvement in swallowing difficulty. Second, different neck postures during each exercise maneuver could affect the RMS value of the swallowing muscles. Before performing each exercise maneuver, however, we attempted to find the muscle belly by using ultrasound. We only measured the RMS value at the end-position of each exercise maneuver to minimize motion artifacts. To investigate the exact contraction of the swallowing muscles, further studies with more detailed protocol of the sEMG measurement may be necessary. Third, we could not evaluate normalization value (%, percent) to control for the variation that can occur for each subject. For sEMG normalization, sEMG were firstly acquired when patients did not perform any swallowing action, which were used as a base line in previous study. To minimize the need for normalization value, however, we analyzed the RMS value of the swallowing muscles in the three different exercises in the same participants. For more accurate analysis, further studies with normalization of sEMG with respect to values measured during resting state may be necessary. Finally, despite the use of sEMG, we could not evaluate all swallowing-related muscles. To investigate the exact contraction of all swallowing muscles, further studies with more swallowing-related muscles may be necessary.

## Conclusion

Considering the superiority in selective contraction and activation of the suprahyoid and infrahyoid muscles, the submandibular push exercise could be an efficient swallowing exercise, as well as the Shaker and CTAR exercises. However, to confirm improvement in actual swallowing difficulty, further studies with more than 6 to 12 weeks of resistance training may be necessary.

## Supplementary information

Supplementary file1 (DOCX 1565 kb)
