# Supplementary material for: Effect of the submandibular push exercise using visual feedback from pressure sensor: an electromyography study
Source: Sci Rep. 2020 Jul 16;10:11772. doi: 10.1038/s41598-020-68738-0 (PMC7366651; doi:10.1038/s41598-020-68738-0)

**Effect of the Submandibular Push Exercise using Visual Feedback from Pressure Sensor: An Electromyography Study**

Sungwon Park^1^, Joo Young Cho^1^, Byung Joo Lee^1^, Jong-Moon Hwang^2^, Myunghwan Lee^3^, Soo Yeon Hwang^3^, KwanMyung Kim^4^, Ki Hoon Lee^5^, Donghwi Park^6^

^1^Department of Rehabilitation Medicine, Daegu Fatima Hospital, Daegu, South Korea

^2^Department of Rehabilitation Medicine, School of Medicine, Kyungpook National University, Kyungpook National University Hospital

^3^Medical Device Development Center, Daegu-Gyeongbuk Medical Innovation Foundation, Daegu, Republic of Korea

^4^Graduate School of Creative Design Engineering, Ulsan National Institute of Science and Technology, Ulsan, Republic of Korea

^5^Mompyeonhan Rehabilitation Clinic, Daegu, Republic of Korea

^6^Department of Physical Medicine and Rehabilitation, Ulsan University Hospital, University of Ulsan College of Medicine, Dong-gu, Ulsan, Republic of Korea

Short title: Effects of submandibular push exercise on dysphagia

Corresponding author: Donghwi Park, M.D. Department of Physical Medicine and Rehabilitation, Ulsan University Hospital, University of Ulsan College of Medicine, 877, Bangeojinsunhwando-ro, Dong-gu, Ulsan, 44033, Republic of Korea Tel.: +82-10-9558-8901 E-mail: bdome@hanmail.net

Funding: None

DISCLOSURE/CONFLICT OF INTEREST: None

**Supplement 1.** The submandibular push exercise and its mechanism for strengthening of supra- and infra-hyoid muscles. Participants were educated to increase submandibular pressure without neck flexion. Using the visual feedback from the pressure sensor on the computer monitor, participants were educated to push the pressure sensor on the submandibular area (by bloating their submandibular area with their lips and teeth closed). Resting state (A)(C), mechanism (B), during submandibular push exercise (D), and a participant wearing headgear with pressure sensor under the suprahyoid muscle (F), chin tuck against resistance (CTAR)(G), Shaker`s exercise (H)

**
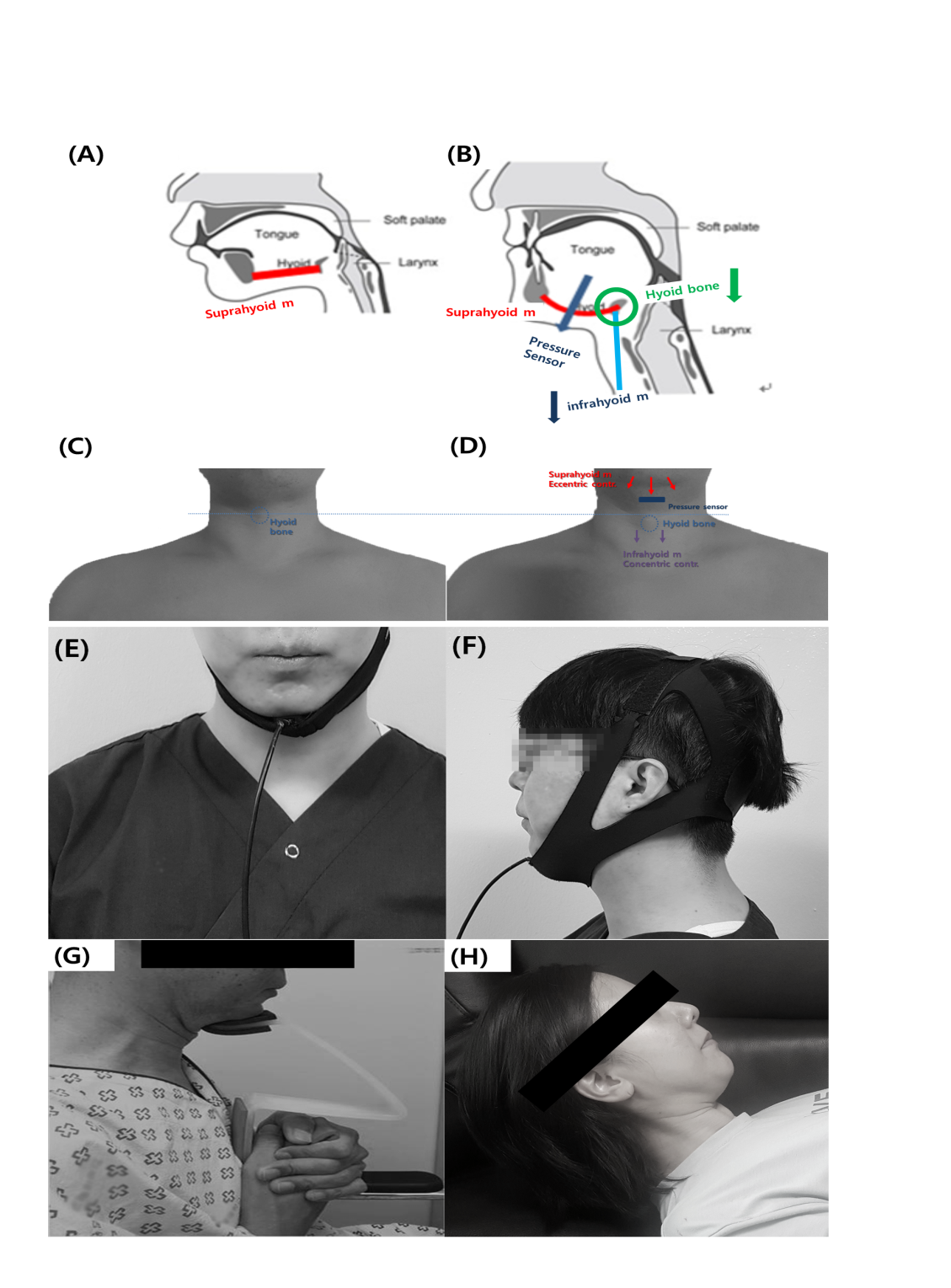
**

**Supplementary 2.** The maximal pressure detected from pressure sensor, and RMS value of the suprahyoid, thyrohyoid, sternohyoid and SCM muscles during submandibular push exercise.

| Name | Age | Sex | Max Suprahyoid RMS (µV) | Max Thyrohyoid RMS (µV) | Max Sternothyroid RMS (µV) | Max SCM RMS (µV) | Max Pressure  (g) |
| --- | --- | --- | --- | --- | --- | --- | --- |
| Subject 1 | 24 | M | 192 | 217 | 169 | 44 | 191.15 |
| Subject 2 | 29 | M | 208 | 114 | 194 | 44 | 226.19 |
| Subject 3 | 28 | M | 75 | 72 | 54 | 30 | 125.52 |
| Subject 4 | 23 | F | 131 | 152 | 217 | 116 | 288.57 |
| Subject 5 | 30 | F | 143 | 179 | 224 | 38 | 195.21 |
| Subject 6 | 27 | M | 186 | 143 | 224 | 38 | 253.57 |
| Subject 7 | 37 | M | 380 | 188 | 264 | 61 | 274.21 |
| Subject 8 | 29 | M | 201 | 260 | 204 | 95 | 177.84 |
| Subject 9 | 39 | M | 446 | 191 | 396 | 45 | 435.42 |
| Subject 10 | 35 | M | 361 | 241 | 295 | 95 | 278.31 |
| Subject 11 | 28 | M | 75 | 72 | 54 | 249 | 250.22 |
| Subject 12 | 29 | M | 72 | 80 | 92 | 45 | 111.5 |

Max; maximal, RMS; root mean square, SCM; sternocleidomastoid, M; male, F; female

**Supplementary 3.** The correlation between the RMS value of the swallowing muscles and the maximal pressure detected from pressure sensor during submandibular push exercise.

|  | | Max Suprahyoid RMS | Max Thyrohyoid RMS | Max Sternothyroid RMS | Max SCM RMS |
| --- | --- | --- | --- | --- | --- |
| Max Pressure (g) | Pearson Correlation coefficients (r^2^) | .753^**^ | .321 | .796^**^ | .156 |
|  | p-value | **.005** | .309 | **.002** | .628 |

Max; maximal, RMS; root mean square, SCM; sternocleidomastoid

** p<0.01, *p<0.05

**Supplementary 4.** The scatter plots of the RMS value of the swallowing muscles and the maximal pressure detected from pressure sensor during submandibular push exercise (A). The correlation between the RMS value of the swallowing muscles and maximal pressure detected from pressure sensor during submandibular push exercise (B-D). Graphs were drawn using IBM SPSS version 21 (SPSS, Inc., Chicago, IL, USA) and PRISM software version 8.00 (GraphPad Software, Inc., San Diego, CA, USA)

**(A)**

**(B-D)**

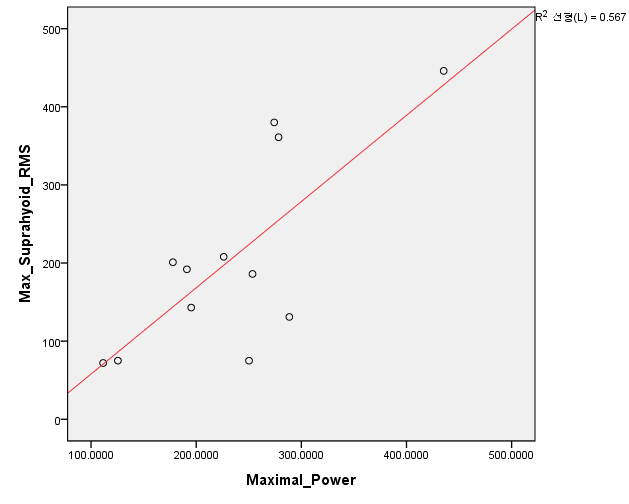

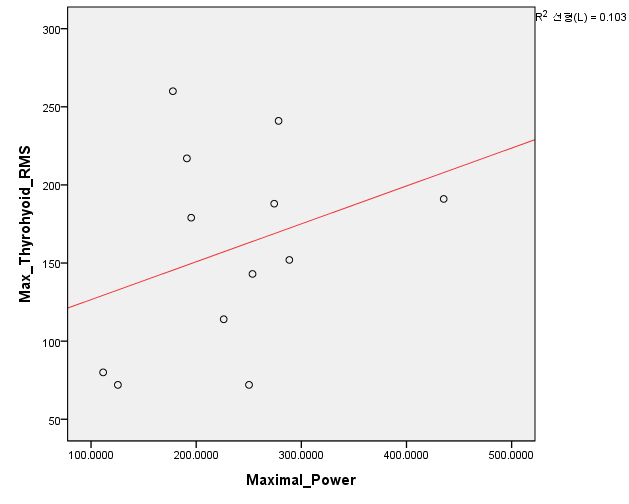

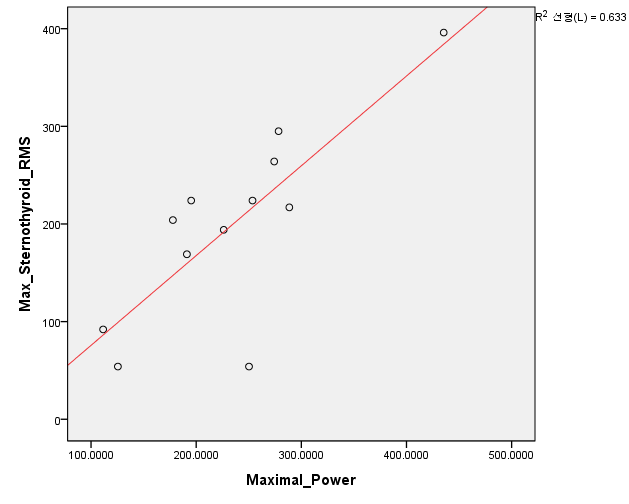


**Supplementary 5.** Figure shows the location of electrodes of surface electromyography in neck area in the study.


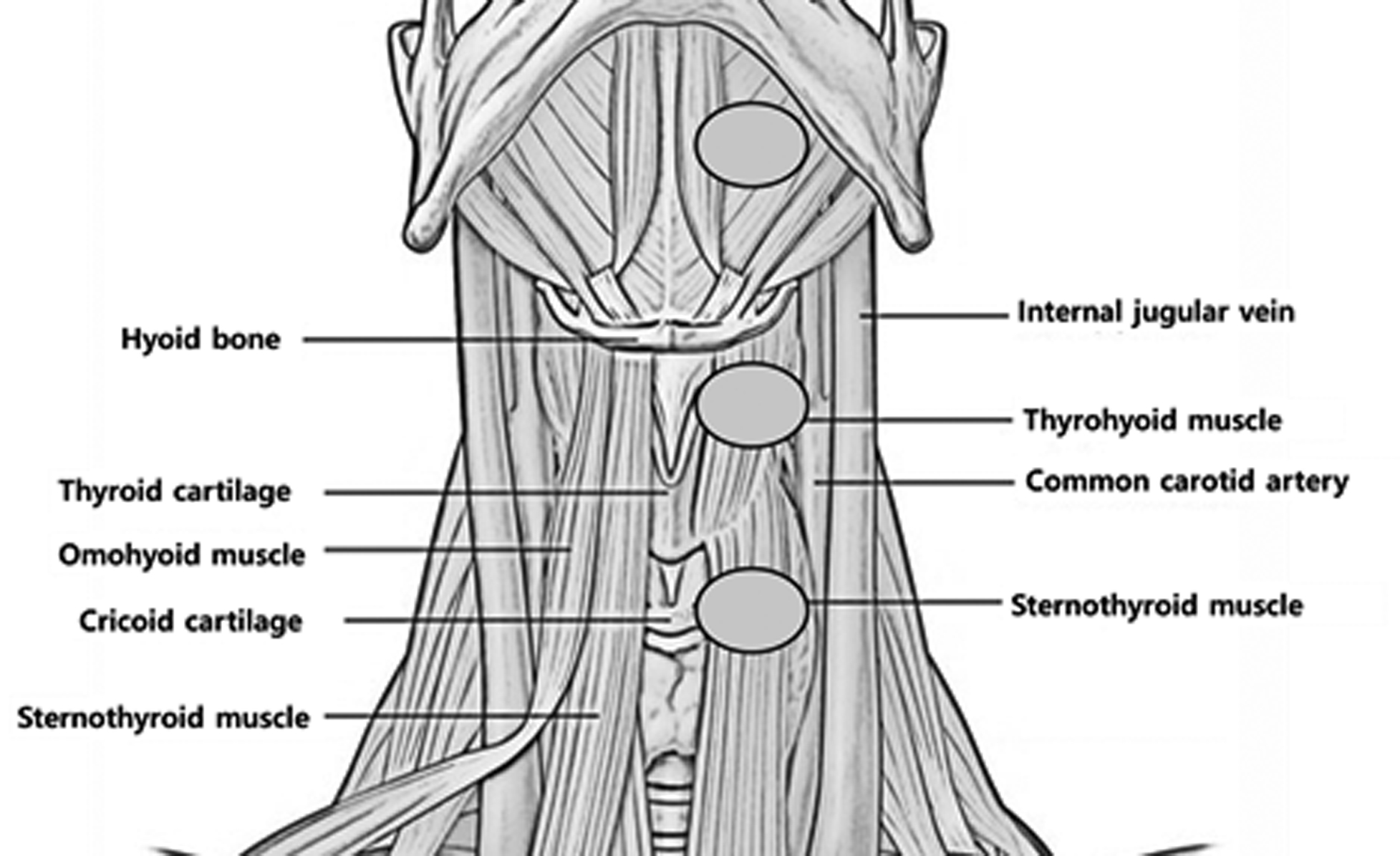


**Supplementary 6.** Figure of tool, such as elastic-plastic bar, which may provide a resistance against suprahyoid muscle during submandibular push exercise.


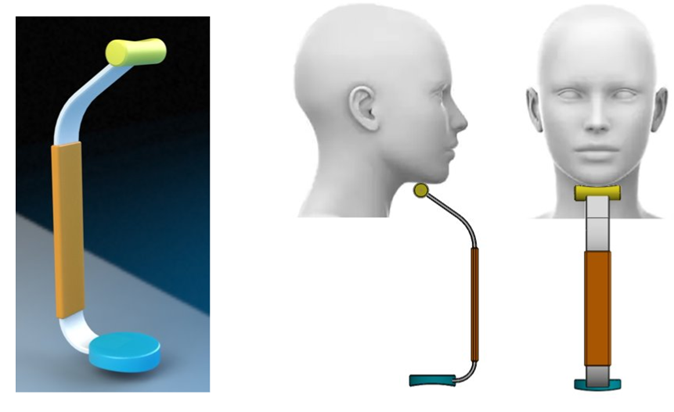

Supplement: Supplementary file 1 — Supplementary file1 (DOCX 1565 kb) [file 41598_2020_68738_MOESM1_ESM.docx]
